# Heavy Water Reduces GFP Expression in Prokaryotic Cell-Free Assays at the Translation Level While Stimulating Its Transcription

**DOI:** 10.1155/2013/592745

**Published:** 2013-12-25

**Authors:** Luisa S. Hohlefelder, Tobias Stögbauer, Madeleine Opitz, Thomas M. Bayerl, Joachim O. Rädler

**Affiliations:** Center for Nanoscience (CeNS), Ludwig-Maximilians-Universität, Geschwister Scholl Platz 1, 80539 Munich, Germany

## Abstract

The *in vitro* proliferation of prokaryotic and eukaryotic cells is remarkably hampered in the presence of heavy water (D_2_O). Impairment of gene expression at the transcription or translation level can be the base for this effect. However, insights into the underlying mechanisms are lacking. Here, we employ a cell-free expression system for the quantitative analysis of the effect of increasing percentages of D_2_O on the kinetics of *in-vitro* GFP expression. Experiments are designed to discriminate the rates of transcription, translation, and protein folding using pDNA and mRNA vectors, respectively. We find that D_2_O significantly stimulates GFP expression at the transcription level but acts as a suppressor at translation and maturation (folding) in a linear dose-dependent manner. At a D_2_O concentration of 60%, the GFP expression rate was reduced to 40% of an undisturbed sample. We observed a similar inhibition of GFP expression by D_2_O in a recombinant *Escherichia coli* strain, although the inhibitory effect is less pronounced. These results demonstrate the suitability of cell-free systems for quantifying the impact of heavy water on gene expression and establish a platform to further assess the potential therapeutic use of heavy water as antiproliferative agent.

## 1. Introduction

Heavy water or deuterium oxide (D_2_O) is a stable isotope of water where deuterium (^2^H) replaces both protium (^1^H) atoms. Natural water comprises a low percentage of deuterium atoms (the natural abundance is about 0.01%) which exchange between adjacent water molecules at frequencies in the THz range. Deuterium bonds can exhibit a higher binding energy and a shorter binding length than protium (hydrogen) bonds. Pure D_2_O (i.e., 99.9% deuterium enrichment) features an 11% higher density, 23% higher viscosity, and 6% higher pD (the equivalent of pH in D_2_O) as compared to water. In life sciences, D_2_O is mainly known as an indispensable solvent and/or isotopic label in proton NMR studies of proteins [1] as well as a unique scattering contrast variation agent in neutron scattering studies of biological molecules [2]. Less well known but highly interesting and complex are its biological effects on intracellular functions [3–5]. The interest in these effects arises because of their potential for a deeper understanding of intracellular processes and the development of novel therapeutical approaches for the treatment of hyperproliferative cell diseases. In biomolecules like enzymes, receptors, DNA, and RNA, an exchange of labile (i.e., exchangeable) protons by deuterium can result in conformational changes [6] due to the fact that deuterium bonds are stronger and shorter than the comparable hydrogen bonds. For cell division and protein expression, this may have two severe consequences: at the transcription level, major enzymes like polymerases with high substrate specificity could be inhibited either directly by D-induced conformational changes or by a D-bond reinforced DNA double strand which may hamper its splitting up into single strands. This would lead to impairment of DNA replication and consequently of cell division and of all protein expressions. At the translational level, reduced mitotic spindle formation [7–9] most likely due to alterations of tubulin conformation by deuterium atoms replacing protons H-bonded to the proteins may cause cell cycle arrest. Furthermore, the (partial) inhibition of protein expression as well as protein misfolding could trigger apoptosis or hamper cell growth and division. Although previous studies characterized the toxicity of deuterium oxide in animals [10–12] by causing severe damages to specific organs such as the mouse kidney [10], deuterium oxide is still believed to have potential as an antiproliferative agent. While deuterium oxide at concentrations greater than 20% of body weight is highly toxic to animals [13], low concentrations of deuterium oxide seem to be harmless for animals and humans, and deuterated intraocular dyes are considered to be safe for clinical use [14]. Furthermore, several studies provided evidence for a significant reduction of human cancer cell lines growth in D_2_O culture medium [15], impairment of human tumor growth in animal models [16, 17], and generally a reduction of cellular proliferation at high deuterium concentrations in prokaryotic and eukaryotic cells [16, 18, 19].

However, there is a notable scarcity of information about the underlying effects of D_2_O on the mechanism of gene expression in the literature. This is surprising as D_2_O may have the potential as an anticancer drug and/or as an adjuvant for established chemotherapeutic drugs. As the human immune system does largely not respond to D_2_O as a foreign molecule, side effects in therapy can be expected to be negligible, a huge advantage over all treatments available.

To obtain further insight into the effects of D_2_O on gene expression in cells, a method is required which can distinguish between transcription and translation in a quantitative way. A cell-free gene expression system can provide this distinction and represents a suitable model system, owing to its reduced complexity, to address this problem [20]. Here all effects revert back directly to the expression machinery due to the absence of downstream repair mechanisms which would obscure the results in cell and tissue cultures.

Here we report on the effect of D_2_O on protein expression using a prokaryotic cell-free system from *E. coli* and a GFP reporter gene. By combining this method with real-time reverse transcription PCR for mRNA quantification [21, 22], each single step of gene expression was analyzed individually. Furthermore, in order to compare the results with a more complex biological *in-vitro* system, GFP expression rates were examined under the influence of D_2_O in a recombinant *E. coli* strain. This provides some quantitative insight into the robustness of the process of gene expression in a living cell.

## 2. Results

**Figure 1:**
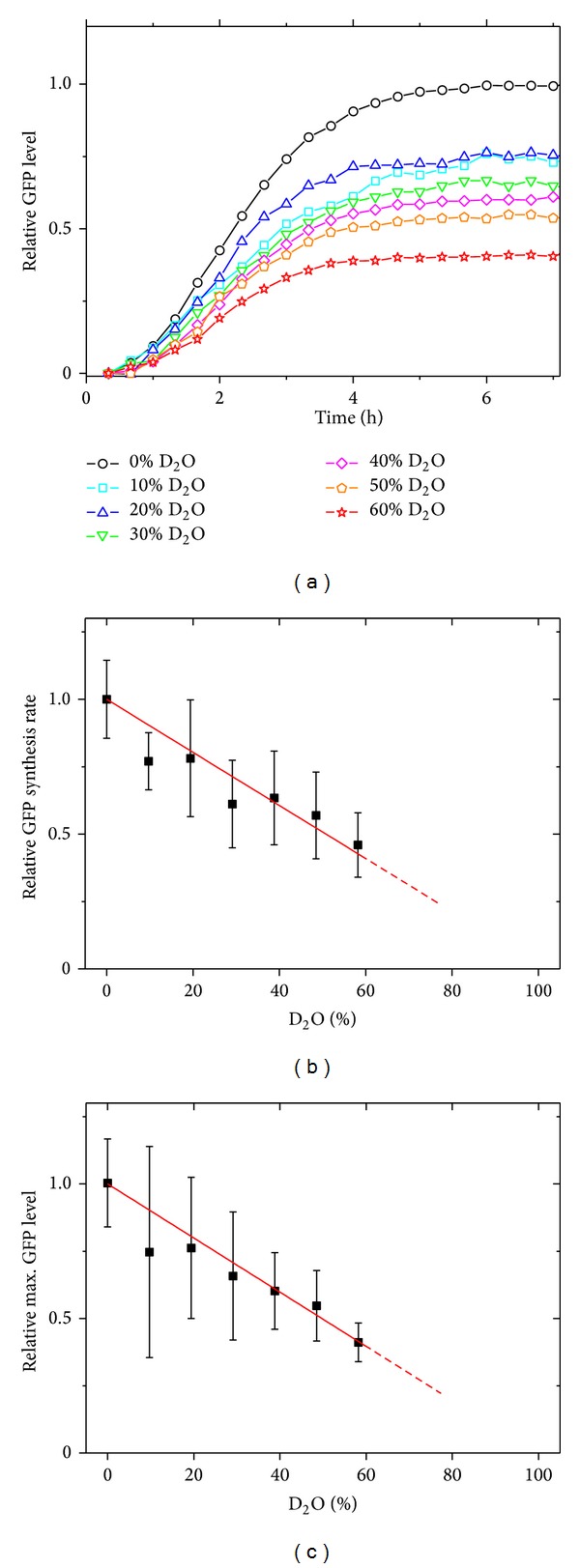
(a) Typical GFP expression kinetics in a cell-free system in the presence of D_2_O (0 to 60%). (b) The GFP synthesis rate (mRNA transcription plus protein translation) is reduced in a deuterated environment. (c) Reduction of GFP synthesis rate results in a smaller yield of GFP when the cell-free synthesis reaction expires after about four to five hours due to degradation of ribosomes.

In a first step, the overall effect of D_2_O on *in vitro* gene expression rates was studied by using a commercial prokaryotic cell-free gene expression system reconstituted of purified components from *E. coli* and a T7 RNA polymerase [20, 23], where H_2_O was replaced by D_2_O. D_2_O concentrations in the samples ranged from 0% (control) to 60% and a fixed amount of plasmid DNA encoding GFP was added to each sample. GFP expression was quantified by fluorescence versus time over 3 hours and maximum GFP yields and expression rates were obtained as described in the methods section. As shown in Figure 1, an approximate linear reduction of both parameters was observed with increasing D_2_O concentration. At a D_2_O concentration of 60%, the GFP expression rate was reduced to 50% and the GFP yield was reduced to 40% compared to the control. A general exchange of H-bonds by D-bonds during GFP expression can take place and affect the fluorescence properties of the GFP molecule if these exchanges are in the region of the fluorophore. As the GFP expression rate is reduced by 50% at a D_2_O concentration of 60%, we believe that changes in the fluorescent properties of the GFP molecule are not sufficient to explain this significant decrease in GFP expression rate. Additionally, the solvent D_2_O might have an indirect effect on the fluorescence properties of the GFP. For a different GFP version (GFP S65T), D_2_O does not alter the absorbance spectra significantly [24, 25]. To verify that this observation holds true for the GFP variant used in this study, we performed a comparative analysis of the fluorescent spectra of the fluorophore diluted in D_2_O and H_2_O (see Supporting Figure 1 in Supplementary Material available online at http://dx.doi.org/10.1155/2013/592745). Our data show that the GFP fluorescence spectrum is not affected by the replacement of H_2_O by D_2_O (Supporting Figure 1), indicating that the fluorescence data shown in Figure 1 are intrinsically related to the effect of D_2_O on the GFP expression.

**Figure 2:**
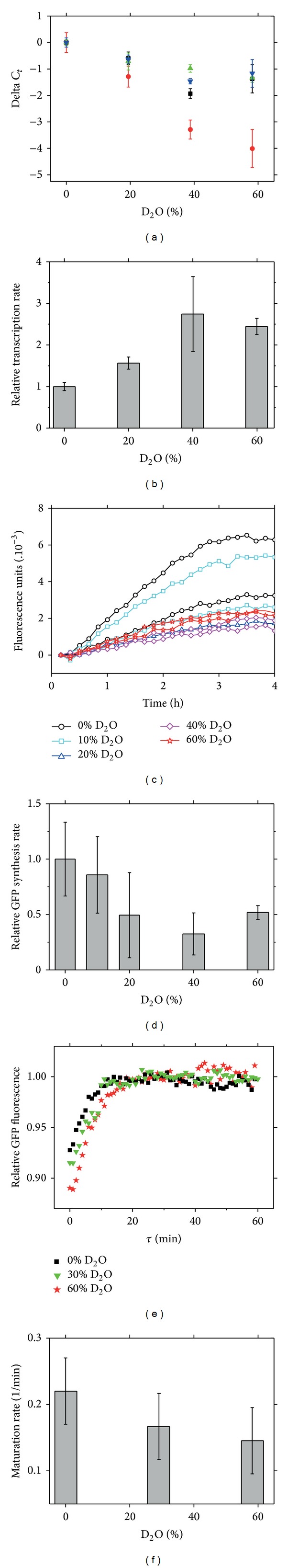
(a) Effect of D_2_O on cell-free transcription. First mRNA was transcribed in a cell-free system with 0%, 20%, 40%, and 60% D_2_O, respectively. Then the mRNA was purified and cDNA was produced using a reverse transcriptase. The amount of cDNA in each sample was subsequently measured by real-time PCR. At 60% D_2_O, the mRNA yield was increased by about a factor of two to three compared to samples with H_2_O. (b) Relative transcription rate in the presence of D_2_O. (c) The effect of D_2_O on GFP translation in a cell-free system. mRNA was transcribed and purified and in a second step used as template in a cell-free system with different D_2_O concentrations. (d) Relative GFP synthesis rate in the presence of D_2_O. (e) Effect of D_2_O on the folding time of GFP. mRNA translation in the cell-free system was stopped by adding the antibiotic chloramphenicol to the sample at the time point *t*_0_ (ribosome deactivation). Any increase in fluorescence after *t*_0_ is due to maturation of already translated GFP. The maturation time *τ* is remarkably longer in D_2_O (66% increase of *τ* at a concentration of 60%) than in H_2_O.

In a second step, we addressed the question at which level of gene expression D_2_O showed the major effect by studying transcription, translation, and protein folding separately (Figure 2).

The transcription efficiency was analyzed by running the cell-free expression kit for 4 hrs in different D_2_O concentrations and subsequent mRNA isolation and purification. To quantify the mRNA yields, one-tube reverse transcriptase real-time PCR using oligo-(d)T primers was applied. The threshold cycles (*C*_*t*_), being inversely related to the amount of amplicon in each sample, were calculated in order to obtain the relative amount of template cDNA, which is directly related to the relative mRNA concentration. Figures 2(a), and 2(b) indicate a nonlinear dose-dependent stimulating effect on the transcription by D_2_O with: Δ_*C*_*t*__  H_2_O > Δ_*C*_*t*__  D_2_O for all D_2_O concentrations studied, where Δ_*C*_*t*__ = 0 represents the undisturbed sample (0% D_2_O). The maximum increase of transcription rate observed at 40% D_2_O amounts to an increase of the mRNA transcription by about 250% compared to 0% D_2_O. At higher D_2_O concentration (60%), the amount of mRNA transcribed levels off.

The effect of D_2_O on translation was studied by using mRNA obtained from cell-free expression in H_2_O. This approach allowed running the transcription step of the experiment under undisturbed conditions. Then, the mRNA obtained from the undisturbed transcription was extracted, purified, and used as template in a new series of cell-free gene expression experiments at varying D_2_O concentrations (0%, 10%, 20%, 40%, and 60%). Translation rate and GFP yield were determined from time-dependent fluorescence measurements as described above. D_2_O significantly inhibits translation in terms of overall GFP yields (Figure 2(c)) and GFP synthesis rates (Figure 2(d)) with the maximum reduction of the rate at 60% (at a D_2_O concentration of 40%) below that of the undisturbed sample. Higher concentrations of D_2_O have no further reducing effect on the *in vitro* translation efficiency.

In a third step, we studied the influence of D_2_O on GFP maturation, which is completely autocatalytic and is not dependent on the presence of molecular chaperones [26–28], by analyzing the process as a function of the GFP folding time. The antibiotic chloramphenicol, which deactivates the ribosomes and thus inhibits translation [29], was added 2 hrs after starting the cell-free expression to ensure robust GFP expression and the GFP signal intensity was recorded. In a correctly folded protein, the GFP fluorophore is localized in the center, where it is protected from the environment. Its fluorescence is solely detectable after this final protein configuration has been adopted. Since the synthesis of new GFP is inhibited after-antibiotic addition, any further fluorescence increase is solely due to maturation of GFP translated under preantibiotic conditions. As shown in Figures 2(e) and 2(f), D_2_O does not inhibit GFP maturation but decreases its maturation rate by 40% (at 60% D_2_O) compared to the control (*τ*_D_2_O_ = 7, 5 min; *τ*_H_2_O_ = 4, 5 min), indicating that the maturation process itself is not affected but significantly slowed down.

**Figure 3:**
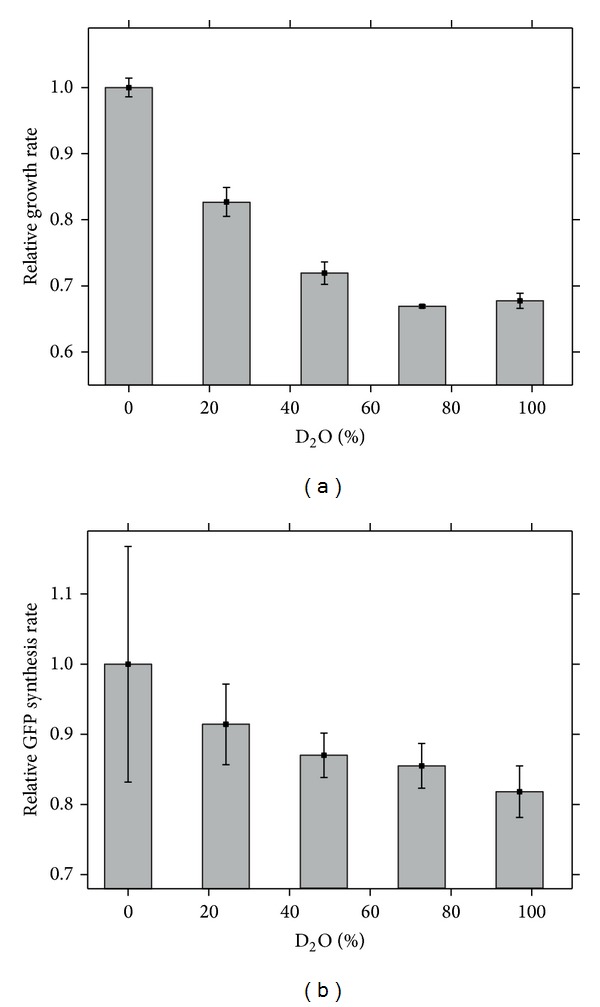
*E. coli* growth rate (a) and GFP expression rate as a function of D_2_O concentration (b).

To compare the results from the simplified model of a cell-free expression system with a complex, but robust biological system containing the whole set of enzymes and molecules that influence gene expression, we analyzed GFP expression by observing its time dependent fluorescence intensity in bacteria (*E. coli*) exposed to M63 minimal media containing different concentrations of D_2_O (total observation time, 5 h).  Figure 3(a) demonstrates an inhibitory effect of D_2_O on bacterial cell proliferation (growth rate) in agreement with results described elsewhere [19]. At the maximum D_2_O concentration of 98%, the cell growth rate was reduced to 65% of the value obtained for the control (i.e., M63 medium prepared solely with H_2_O). Interestingly, the growth rate in nonlinear with the D_2_O concentration with the major growth inhibitory effect is observed for D_2_O concentrations up to 50%, while at higher concentrations the effects level off.  Figure 3(b) shows that the effect of D_2_O on the GFP synthesis rate in *E. coli* is less pronounced but approximately linear: at the maximum concentration of D_2_O (98%), the synthesis rate is down to 83% of the control value.

## 3. Discussion

Heavy water is quite obviously a very peculiar modulator of cellular activity: it can enter the cell (or even a whole organism) quickly and undetected by any of the cellular mechanisms which would kick in for any other small molecules invading the cell. This is mostly because it passes as normal water and can use its entry (and exit) passages. Inside the cell, it readily enters all organelles, including nucleus, nucleolus, and the endoplasmic reticulum, in the same way water does, where it can cause severe damages to theses organelles [10]. In particular, it can get in contact with all hydrophilic parts of the cellular proteins and DNAs allowing the deuterons to exchange with their labile protons. Note that the speed of the D_2_O distribution in the cytoplasm is not limited by the self-diffusion constant of the water molecule as such but rather by the “atomic” diffusion of the deuterons which is at least one order of magnitude faster. This is because the deuterium exchange rate between individual oxygen atoms of the water is very fast (picosecond-range), giving in the time average H–O–D rather than H_2_O and D_2_O. This behavior can also explain why it can pass readily through cellular and intracellular barriers, in particular membranes.

Most studies in the literature largely concentrated on the quantitative effects of D_2_O on intracellular processes like cell division and proliferation without providing clues about the underlying physiological and biophysical mechanisms. However, it is essential to elucidate these processes in order to devise strategies on if and how D_2_O could be used in humans for the treatment of hyperproliferative organ diseases like tumors. Our results present a first step in this direction by studying the question whether the intracellular D_2_O effects on gene expression weight more on transcription, translation, or maturation of proteins. Here the combination of a commercially available cell-free expression system and standard real-time PCR provided a powerful tool kit to address this problem.

Our observation that D_2_O significantly stimulates transcription in the cell-free assay (Figures 2(a) and 2(b)) is surprising as it indicates that D_2_O does not solely act as a decelerator of protein activity but rather can accelerate a complex process like DNA transcription and thus increase the yield of mRNA. A rationale of this acceleration of an enzymatic reaction can only be surmised here based on the knowledge that deuterons alter hydrogen bonds: D_2_O may modify a rate-limiting step in the separation of the two DNA strands via the helicases by a change of the binding strength between the two strands by means of replacing H-bonds by D-bonds [6, 30]. Another mode of action could be improvement of enzymatic activity via stabilization of their structure [4], induction of slight changes of their folding, or by the way the enzymes bind to the DNA strand. It would be interesting to learn if a further increase of the D_2_O concentration beyond the upper limit of 60% as covered in the present study would push transcription efficiency even higher and where the effect may level off or reverse. However, technical limits need to be overcome prior to such a study and we will focus on this question in a separate investigation.

**Figure 4:**
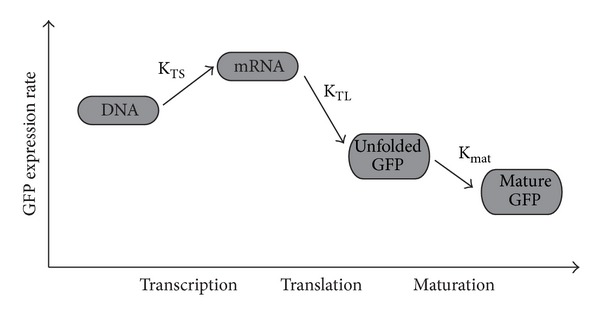
Schematics of the observed effect of D_2_O on transcription, translation, and GFP maturation in the cell-free protein synthesis system. Transcription efficiency was found to increase, whereas translation and maturation efficiencies were diminished. The observed overall effect of D_2_O on the GFP expression rate is negative (see text for details).

As we observed that overall gene expression is markedly slowed down by D_2_O (Figure 3) for the fully functional cell, we can assume that either translation and/or maturation were decelerated in the presence of D_2_O. Indeed, our cell-free assay results indicate that both steps were similarly slowed down by D_2_O as schematically depicted in Figure 4. For translation, we observed the strongest deceleration with increasing D_2_O concentration (Figures 2(c) and 2(d)). Exchange of the solvent H_2_O by D_2_O did not alter the pH significantly (Supporting Table 1), and we can therefore rule out effects of D_2_O on the pH as a major cause reducing the translation rate. Exchange of H-bonds by D-bonds during translation and/or the following maturation might affect the fluorescent properties of the GFP molecule, if such an exchange is taking place in the region of the fluorophore. But as the GFP synthesis rate drops down by more than 50%, this explanation does not seem to be sufficient. A major cause could be changes in the viscosity [31–33], which is increasing with the D_2_O concentration: at 60% D_2_O, the viscosity is increased by 13.8% as compared to pure water, potentially affecting the interaction between the ribosome and the mRNA as well as amino acids. Furthermore, one may speculate that the ribosomal machinery is slowed down in a rate-limiting step by changes connected with the partial replacement of H-atoms by deuterons in this multisubunit protein complex. The importance of water and its hydrogen-bonding networks for ribosomal function was reported by [34] which represents a major factor of the ribosomal entropic stabilization [35–37].

In the cell-free system employed for this study, maturation is essentially represented by self-contained folding of the GFP, while no chaperones are involved. Hence, the slowing down of maturation (Figures 2(e) and 2(f)) indicates that the GFP takes longer time to attain its final folding structure, possibly owing to the presence of stronger D-bonds which may require more time to break for reaching the protein's final conformation. It is well known that GFP folding comprises some dehydration steps [26–28], where a stronger D-bond may require more time to break by thermal forces. This result indicates that chaperone inhibition by D_2_O, which was suggested previously as a major contributor to D_2_O induced overall slowing down or inhibition of viral gene expression [38] in cells, is rather unlikely to represent an essential factor.

It appears not surprising that we observed for the fully functional cell a reduction of gene expression being less sensitive to the D_2_O concentration compared to the cell-free assay. After all, the cell has a wide range of tools available to minimize the effect, in particular DNA/RNA proofing and repair mechanisms, while the T7 polymerase used in the cell-free assay is unable to perform corrections. For a mutant Chlorella algae it was recently shown [39] that gene expression becomes less sensitive to the presence of high D_2_O concentrations by the overexpression of certain heat shock proteins (Hsp60 and Hsp70). This suggests that misfolding and prolonged maturation of proteins may become a more important factor after repair mechanisms at the transcription and translation level kicked in.

It is interesting to note that the *E. coli* growth rate (Figure 3(a)) exhibits a higher sensitivity to the D_2_O concentration in the lower concentration range (0–60% D_2_O) than the GFP expression rate (Figure 3(b)). Here the growth rate reaches a minimum already at 50–60% D_2_O concentration and does not decrease further, while GFP expression decreases linearly over the whole concentration range (0–100% D_2_O). This may indicate that D_2_O has a particular effect on some proteins and/or their expression involved in cell division.

## 4. Conclusions

Cell-free systems enable the analysis of the effects of different molecules on gene expression in a quantitative way in each substep. For D_2_O, the results suggest that the stimulation of the GFP transcription is insufficient for a higher output because the downstream steps (translation and maturation) are hampered. Further studies will shed light on the molecular base of the D_2_O effect on each of the different substeps which may provide new clues for the use of D_2_O as a therapeutic drug if applied at low concentrations.

## 5. Materials and Methods

### 5.1. Plasmid DNA

The gfpmut1 gene [40] was cloned into the pET 23b vector (Novagen, USA) between the T7 promoter and terminator sequence. pET 23b was purified and stored in deionized water at −20°C.

### 5.2. Cell-Free System

#### 5.2.1. Sample Preparation

The commercial cell-free system PURExpress was ordered from New England Biolabs (Frankfurt, Germany) and used as described in the manual. Aliquots of PURExpress components “A” and “B” were stored at −80°C and thawed on ice directly before use. Components were mixed and pET 23b DNA was added in sufficient quantity to ensure saturation of the transcription/translation apparatus [20]. Samples were diluted in H_2_O, D_2_O, or a mix of both to the desired D_2_O concentrations as mentioned in the text. The mixtures of D_2_O and H_2_O were prepared by diluting D_2_O into H_2_O. The final D_2_O/H_2_O mixtures were checked for their pH using color-fixed indicator sticks (Macherey-Nagel) sensitive for different pH ranges. The according pH values can be found in Supporting Table 1. Samples were diluted more than recommended by the manufacturer in order to achieve high D_2_O concentrations, leading to decreased yields of GFP. As a consequence, D_2_O concentrations above 60% were not studied because the dilution required would render the GFP yields below the detectability level. Typical sample volume was 12 *μ*L.

#### 5.2.2. mRNA Purification

pET 23b plasmid and PURExpress were mixed and kept at 37°C for 3 hrs. Subsequently the “RNA cleanup” protocol of the RNeasy Mini Kit (Qiagen, USA) was applied to purify mRNA, which was used as template for cell-free “translation only” measurements.

#### 5.2.3. cDNA Production with Reverse Transcriptase

Cell-free reactions with the desired D_2_O concentrations were run and mRNA was purified as described above. SuperScript II Reverse Transcriptase (Invitrogen, USA) was used to produce cDNA for downward real-time PCR measurements.

#### 5.2.4. Data Acquisition

GFP synthesis was measured in a FLUOstar optima microplate reader (BMG LABTECH) using a 96-well plate and the top optics option. Excitation/emission filter was at 485 nm/520 nm. The temperature control unit was used to keep the samples at a constant 37°C during measurements. The 96-well plates were covered with a Breath-Easy foil (Diversified Biotech, USA) to prevent sample evaporation.

#### 5.2.5. Data Analysis

Plate reader data were analyzed with the Origin 8.5 G data analysis software. Maximum slopes of GFP expression versus time were determined by fitting a sigmoidal curve to the raw data and taking the derivative giving the expression rate. The GFP yield was determined from the plateau of the fluorescence versus time curve and was expressed as a percentage of the undisturbed system (0% D_2_O = 100% GFP yield). Error bars shown in the figures represent the standard deviation at the individual measurement points calculated in Origin 8.5 G. Outliers were omitted from the statistics.

### 5.3. Real-Time PCR

#### 5.3.1. Sample Preparation

PCR sample volume was 50 *μ*L. Each sample consisted of 2 *μ*L DNA, 2 *μ*L of forward (5′-CGC CAC CAT GGT GAG CAA GG-3′) and reverse (5′-GGT TGT CGG GCA GCA GCA CG-3′) primer each, 10 mM dNTPs, 2 *μ*L Sybr Green, 2 U Taq DNA polymerase (New England Biolabs, Germany,) and 0.5 *μ*L of 10× reaction buffer. The remaining 36.5 *μ*L was filled with a D_2_O/H_2_O mix to the desired D_2_O concentration as indicated in the text.

#### 5.3.2. Data Acquisition

Real-time PCR was performed on a C1000 thermal cycler equipped with the CFX96 Real-time Detection System (Bio-Rad, Germany). Reaction conditions were first denaturation at 95°C for 5 min. This was followed by 30 steps of denaturation (94°C, 30 s), annealing (65°C, 30 s), and extension (72°C, 30 s). Final extension was performed at 72°C for 5 min. Analysis of gene expression using real-time PCR showed unique melting curves without primer dimers. The identity of PCR products was verified on a 1.5% agarose gel (data not shown).

#### 5.3.3. Data Analysis

During the exponential phase of PCR, the amount of target DNA doubles with each cycle. The cycle number at which a sample crossed a manually defined threshold (called the *C*_*t*_ value) is therefore indirectly proportional to the amount of template. Real-time PCR data were analyzed with the Origin 8.5 G data analysis software. Sample curves were plotted with logarithmic *y*-axis, a threshold slightly above the background level was defined, and the *C*_*t*_ values were recorded. Outliers were omitted from the statistics.

### 5.4. *E. coli*

#### 5.4.1. Sample Preparation

M63 medium was prepared according to [41]. The pH was adjusted to 7.0 using KOH. The medium was sterilized in an autoclave. The following additions were added to the medium: MgSO_4_ × 7H_2_O (1 M), thiamin (1 *μ*g/mL), casein hydrolysate (0.2%), glycerol (0.2%), ampicillin (1000 ug/mL), and arabinose (0.2%) for maximal fluorescence induction from the inducible promoter pBAD [42]. Aliquots of M63 medium containing pure H_2_O, 25%, 50% 75%, and 98% D_2_O, respectively, were prepared. An overnight culture of *E. coli* (BZB1011-pBAD24-GFP) was grown in M63 with pure H_2_O. The optical density of the culture was checked photometrically (OD = 2.3). 0.5 mL aliquots of M63 medium were filled into a 48-well plate. 22 *μ*L of overnight culture was added to each well, resulting in OD = 0.1 in the samples. Preparation was done in a 3-fold redundancy for each D_2_O concentration for improved data accuracy.

#### 5.4.2. Data Acquisition

Growth rate and GFP expression of *E. coli* as a function of D_2_O content of the medium were measured in a FLUOstar optima microplate reader (BMG labtech). Every 15 min, the absorbance and GFP fluorescence of each well was detected. The plate was kept at a constant 37°C during measurement. 1 h after adding the overnight culture to the 48-well plate, GFP expression was triggered by adding 5 *μ*L of 20% arabinose solution to the wells.

#### 5.4.3. Data Analysis

Data were analyzed using the Origin software. Individual data were averaged omitting possible outliers. For the determination of GFP expression time courses, the GFP fluorescence data were divided by the absorbance data in order to normalize the number of cells over time.

## Supplementary Material

Our fluorescence data indicate that D2O affects the cell-free GFP expression rate. However, D2O might also have an indirect effect on the fluorescent properties of the GFP fluorophore. A general exchange of H-bonds by D-bonds during GFP expression can take place and affect the fluorescence properties of the GFP molecule if these exchanges are in the region of the fluorophore. Additionally, the solvent D2O might have an indirect effect on the fluorescence properties of the GFP. Therefore, a comparativ analysis of the fluorescent spectra of GFP diluted in H2O and D2O was performed. Additionaly, since GFP fluorescence is pH sensitive, it was verified that exchange of the solvent H2O by D2O did not alter the pH significantly. The data show that the GFP fluorescence spectrum is not affected by the replacement of H2O by D2O, indicating that our observed fluorescence data are intrinsically related to the effect of D2O on the cell-free GFP expression.
