# Supplementary material for: Heavy Water Reduces GFP Expression in Prokaryotic Cell-Free Assays at the Translation Level While Stimulating Its Transcription
Source: Biomed Res Int. 2013 Dec 25;2013:592745. doi: 10.1155/2013/592745 (PMC3886400; doi:10.1155/2013/592745)
Supplement: Supplementary file 1 — Our fluorescence data indicate that D2O affects the cell-free GFP expression rate. However, D2O might also have an indirect effect on the fluorescent properties of the GFP fluorophore. A general exchange of H-bonds by D-bonds during GFP expression can take place and affect the fluorescence properties of the GFP molecule if these exchanges are in the region of the fluorophore. Additionally, the solvent D2O might have an indirect effect on the fluorescence properties of the GFP. Therefore, a comparativ analysis of the fluorescent spectra of GFP diluted in H2O and D2O was performed. Additionaly, since GFP fluorescence is pH sensitive, it was verified that exchange of the solvent H2O by D2O did not alter the pH significantly. The data show that the GFP fluorescence spectrum is not affected by the replacement of H2O by D2O, indicating that our observed fluorescence data are intrinsically related to the effect of D2O on the cell-free GFP expression. [file 592745.f1.pdf]

Supporting Information for:

Heavy water reduces GFP expression in prokaryotic cell-free assays at the translation level while stimulating its transcription

Luisa S. Hohlefelder[1≠], Tobias Stögbauer[1≠], Madeleine Opitz[1],
Thomas M. Bayerl, Joachim O. Rädler[1]

[1] *Center for Nanoscience, CeNS, Ludwig-Maximilians-Universität,*
Fakultät für Physik, Geschwister Scholl Platz 1, 80539 München, Germany
[≠] *contributed equally to this paper and should be considered as co-first authors*

Supporting Figures:

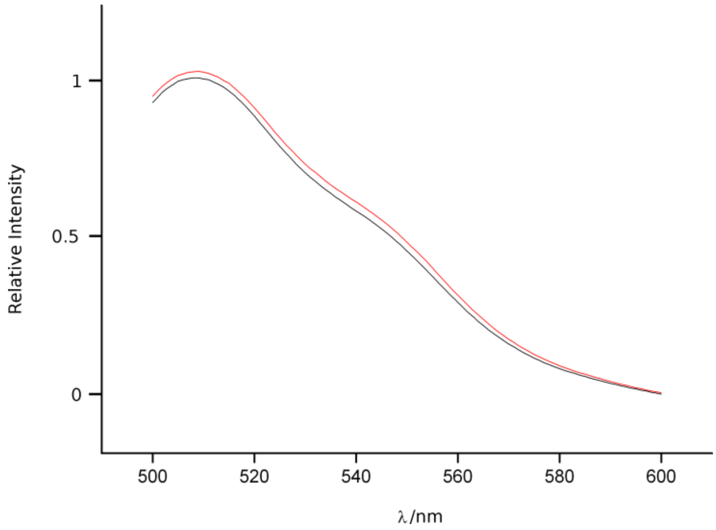

Supporting Figure 1: Emission spectra of GFP diluted in H_2O or D_2O. Black line: GFP fluorescence emission spectra for GFP diluted in H_20. Red line: GFP fluorescence emission spectra for GFP diluted in D_20. Mature/purified GFP was diluted one to ten in H_2O or D_2O respectively and the fluorescence emission spectra were recorded. As can be seen, the GFP fluorescence spectra are quantitatively and qualitatively identical for GFP diluted in H_2O and D_2O. This result is in accordance with previous findings for e.g. the GFP variant S65T. Here, again the observed absorbance spectra of the GFP in H_2O or D_2O are identical [24, 25]. We are therefore confident, that the increase of the relative GFP level in **Fig. 1** is mainly due to a direct effect of D_2O on GFP expression.

Supporting Tables:

Table 1: pH values of the D_2O-H_2O mixtures used in this study

D_2O concentration	pH value
0%	6.98 ± 0.06
10%	6.99 ± 0.07
20%	7,08 ± 0,17
30%	7,08 ± 0,12
40%	7,25 ± 0,20
50%	7,28 ± 0,19
60%	7,53 ± 0.08
